# The Impact of Cues on Joint Attention in Children with Autism Spectrum Disorder: An Eye-Tracking Study in Virtual Games

**DOI:** 10.3390/bs14100871

**Published:** 2024-09-25

**Authors:** Lili Liu, Shuang Li, Lin Tian, Xinyu Yao, Yutao Ling, Jingying Chen, Guangshuai Wang, Yang Yang

**Affiliations:** 1 National Engineering Research Center of Educational Big Data, Central China Normal University, Wuhan 430079, China; liulili@mail.ccnu.edu.cn; 2 National Engineering Research Center for E-Learning, Central China Normal University, Wuhan 430079, China; 3 Faculty of Artificial Intelligence in Education, Central China Normal University, Wuhan 430079, China; lis526@mails.ccnu.edu.cn (S.L.); lintian@mails.ccnu.edu.cn (L.T.); yaoxinyu031962@mails.ccnu.edu.cn (X.Y.); yangslin@mails.ccnu.edu.cn (Y.Y.); 4 College of Physical Science and Technology, Central China Normal University, Wuhan 430079, China

**Keywords:** children with ASD, joint attention, virtual reality, eye tracking

## Abstract

Joint attention (JA), a core deficit in children with autism spectrum disorder (ASD), is crucial for social interaction, emotional understanding, and cognitive development. This study aims to compare and analyze the eye-tracking data of ASD and typically developing children (TDC) during virtual games, exploring how different cue types affect JA performance in ASD children. A total of 31 TDC and 40 ASD children participated in the study. Using eye-tracking devices, we recorded the children’s eye movements as they played virtual games, selecting the correct target based on cues provided by virtual characters. Our findings revealed that different cue types significantly impacted the game scores of ASD children but had no significant effect on TDC, highlighting a notable disparity between the two groups. ASD children showed a lower fixation frequency, irregular fixation paths, and increased attention to non-target objects compared to TDC. Interestingly, among the three cue types, ASD children exhibited a preference for the third type, leading to longer fixation on the region of interest and higher game scores. These results underscore the importance of cue selection in enhancing JA in ASD children. This study provides novel insights into the JA deficits in ASD children and offers a scientific basis for the development of targeted and individualized intervention programs.

## 1. Introduction

Autism spectrum disorder (ASD) is a neurodevelopmental disorder characterized by communication disorders, difficulties in social interaction, and restricted, repetitive patterns of behavior [1]. In recent years, the incidence of autism has continued to rise. According to the latest statistics from the Centers for Disease Control and Prevention (CDC) in 2023, one in every 36 (2.76%) eight year olds is confirmed to have ASD [2]. The change in incidence from 1:44 to 1:36 indicates that the rehabilitation field is facing increasingly severe challenges due to the sharp increase in the number of children with ASD.

In the ASD population, the severity and specific manifestations of social cognitive impairment vary significantly. ASD individuals may have mild to severe impairments in understanding and responding to social cues, which are typically manifested in defects in joint attention (JA) [3,4]. JA refers to the ability of two or more individuals to simultaneously focus on the same object or event, and it serves as the foundation for social cognitive development, facilitating the development of various advanced functions such as language acquisition and emotional understanding [5,6,7]. Typically developing children (TDC) begin to exhibit rudimentary JA abilities as early as six months old, such as following the gaze of others [8]. In contrast, ASD children often exhibit significant delays in these skills, leading to difficulties in social interaction and communication [9].

Interpreting and responding to social cues, particularly eye contact, is crucial for effective social cognition [10,11]. Eye contact provides individuals with vital information about others’ focus of attention and intentions [12], which are central elements in social interactions. Dalmaso (2022) points out that eye contact plays a pivotal role in social cognition, enabling individuals to gather information about others’ emotional states, intentions, and focus of attention [13]. This process is essential in understanding and predicting social behaviors, particularly when studying populations with social cognitive deficits, such as individuals with ASD. Additionally, Johnson et al. (2016) emphasize that investigating eye movement in ASD children offers valuable insights into their unique means of processing social information [14]. Research has shown that ASD children often exhibit atypical eye movement patterns, such as reduced attention to faces and eyes [15,16,17], which may hinder their ability to engage in social interactions. These atypical patterns are not merely symptoms but also reveal the cognitive processes underlying social deficits in ASD [18]. By studying eye movements, researchers can gain a deeper understanding of the specific challenges faced by ASD children and develop interventions that better cater to their needs.

Although a significant amount of research has focused on the development of JA abilities in infants with ASD, relatively few studies have been conducted on school-aged children with ASD. Nevertheless, the school-age period is typically a crucial time for children to refine their social skills [19], and deficiencies in JA during this stage can lead to long-term challenges in social interaction and communication. Therefore, interventions during this phase are vital for the comprehensive development of children with ASD. Additionally, research indicates that improvements in JA are closely linked to academic achievement and peer relationships, suggesting multifaceted long-term benefits of JA interventions [20]. Consequently, systematic and sustained JA interventions are particularly important during the school-age period, as they not only help ASD children to better adapt to their current social environments but also lay a solid foundation for their future social functioning.

Current JA intervention methods for ASD children include traditional interventions, robot-assisted interventions, and virtual reality (VR) technology. Traditional interventions emphasize the development of JA through face-to-face social interactions and professional guidance. For instance, in group interventions, instructors provide structured guidance such as explanations, demonstrations, prompts, scripts, reinforcement, feedback, and rehearsals, which are particularly beneficial for high-functioning children [21]. For low-functioning children, early interventions based on joint control or the Early Start Denver Model use structured activities to build foundational social cognitive skills [22,23]. However, traditional methods may face resource constraints and shortages of professionals, which can result in many children not receiving timely interventions. Robot-assisted interventions utilize robots as interactive partners to create controlled environments, demonstrating potential to enhance social skills and JA in ASD children [24]. Nevertheless, these interventions often require human supervision [25], may have limited effectiveness for certain ASD subgroups, and raise safety concerns [26].

In contrast, VR technology, as an emerging intervention method, demonstrates unique advantages. VR technology can provide controlled virtual environments and personalized interactive modes, enabling ASD children to practice and enhance JA in safe virtual scenarios [27,28]. This study integrates elements from previous face-to-face therapies into a virtual gaming environment, effectively supporting the development of JA in ASD children across different developmental stages through simulated structured guidance and feedback. Additionally, by incorporating eye-tracking technology, VR can precisely measure how ASD children attend to social cues during interactions [29,30,31,32,33]. This integration bridges the gap between understanding the cognitive processes behind eye movement and improving JA interventions.

Despite the potential of these technologies, there is still a lack of research on how different types of cues in VR environments affect the JA performance of children with ASD. In previous studies on interventions aimed at improving the JA skills of children with ASD, few have collected eye movement data from these children, which has prevented a comprehensive analysis of their gaze characteristics under various cues. By combining VR technology with eye-tracking technology, this study is the first to systematically explore the effects of different types of cues on the JA of children with ASD. We not only analyzed the children’s performance in the game but also delved into their preferences for different cues, thereby filling the gaps in existing research. Our study aims to explore the influence of different cues (“gaze”, “gaze with head turn”, and “gaze with head turn and finger pointing”) on the JA performance of children with ASD in virtual games, and to compare these effects with TDC, with the goal of providing a scientific basis for the design of more targeted and personalized intervention programs for children with ASD.

Based on this, the following three key questions are proposed in this study: (1) Are there significant differences in JA performance between children with ASD and TDC in VR games? (2) Do different types of cues have a significant impact on the VR game scores of children with ASD and TDC? (3) Do children with ASD exhibit a significant preference for specific types of cues during the gaze process? By answering these questions, this study aims to provide a scientific basis for the development of more targeted and personalized JA interventions, ultimately leading to better treatment outcomes for children with ASD.

## 2. Materials and Methods

### 2.1. Participants

A total of 71 age-matched children aged between 6 and 9 years participated in this experimental study. Among them, 40 were children with ASD (31 boys and 9 girls, with an average age of 7.05 years), and the remaining 31 were TDC (15 boys and 16 girls, with an average age of 7.06 years) (Table 1). The two groups of participants were recruited from nearby special education institutions and kindergartens, respectively. All participants demonstrated good acceptance of the virtual game based on gazing at the computer screen, with no reports of discomfort.

All children with ASD were diagnosed by two child development and behavior experts in a double-blind manner and met the criteria for ASD in the Diagnostic and Statistical Manual of Mental Disorders. All TDC were assessed for their developmental behavior level using the 0–6 years old Child Development Scale [34], and all children had a developmental quotient exceeding 80 points (reference range for developmental quotient: 130 points or above is excellent, 110–129 points is good, 80–109 points is moderate, 70–79 points is borderline low, and below 70 points is indicative of mental deficits). To ensure comparability between the two groups of children in key demographic variables, special attention was paid to age and gender matching when recruiting the participants.

Furthermore, through interviews with teachers and clinical observations, we confirmed that none of the participants had received any prior intervention related to JA or participated in similar experimental studies before the experiment. Therefore, the potential impact of prior interventions on the experimental results could be excluded. Meanwhile, to control for potential subgroup differences, we aimed to reduce their influence on the results through rigorous participant screening and experimental condition control. To protect the privacy of the participants, we only collected anonymous data related to the completion of the experimental tasks and signed privacy protection agreements and informed consent forms with relevant institutions and the parents of the participants.

### 2.2. Materials and Apparatus

#### 2.2.1. Materials

**Figure 1:**
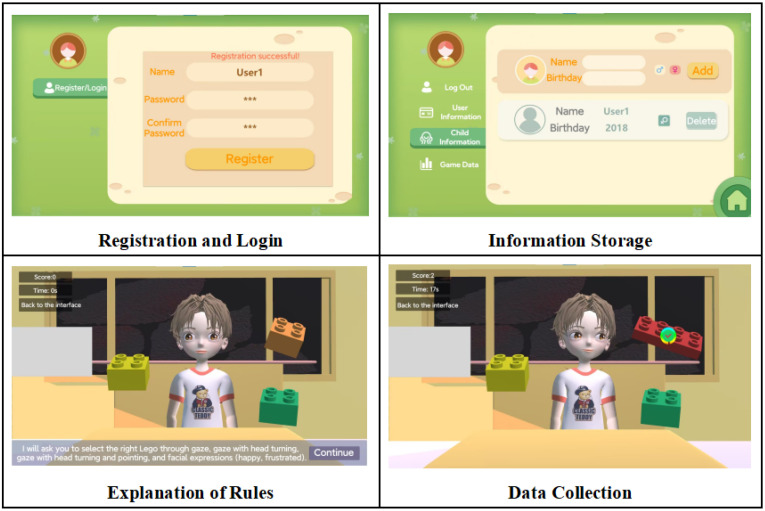
Game interface.

**Figure 2:**
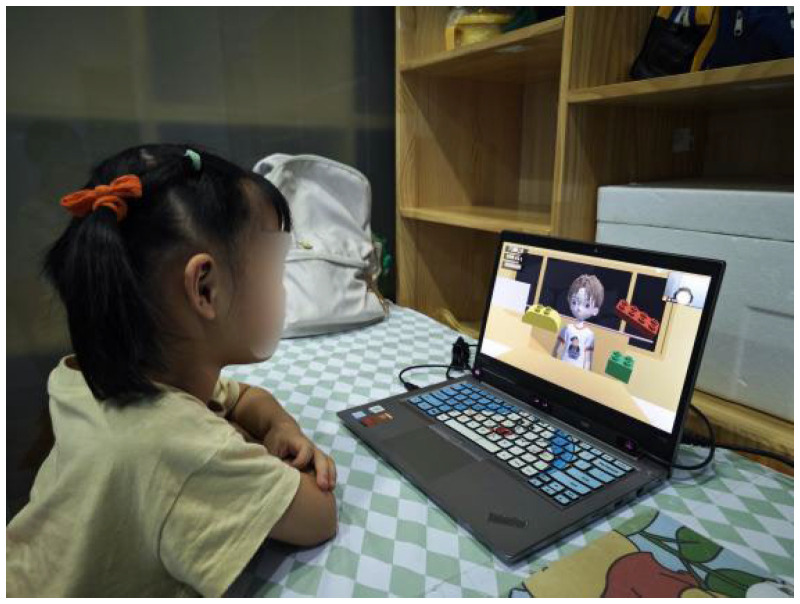
Example of testing environment and procedure.

In this study, we designed a virtual game consisting of the following modules: (1) user registration and login module—for the registration of participants’ basic information, such as their name, birth date, and gender; (2) information storage module—to save participants’ basic information; (3) game rule introduction module—where a virtual character introduces the game rules to the participants; (4) data collection module—to record and save information on participants’ game scores, time, and eye-tracking data. The name of this virtual game was “LEGO Puzzle”, and Figure 1 shows the interfaces of the game’s relevant modules, including the types of cues, the image of the virtual character, and the LEGO elements. The virtual character provides participants with three types of cues: “gaze” (hereinafter referred to as Cue 1), “gaze with head turn” (hereinafter referred to as Cue 2), and “gaze with head turn and finger pointing” (hereinafter referred to as Cue 3), as shown in Figure 2. These cues are presented to participants in a random order during the game. The choice of gaze cues was made due to their proven importance as social signals, which can convey information about the social characteristics of observers, as supported by Dalmaso et al. (2020) [35]. Similarly, head turns and pointing gestures were selected because they are known to effectively shape attention, influencing the direction and focus of an observer’s attention [36]. The basis for selecting these cues lay in their proven relevance in social cognition, particularly in the context of ASD, where the integration and interpretation of such cues are often impaired.

#### 2.2.2. Apparatus

The virtual game was presented on a computer with a screen resolution of 1920 × 1080 pixels using the Unity software (version 2022.1.20f1c1). During the experiment, we used the Tobii Eye Tracker 5 from the Tobii Company in Stockholm, Sweden, to record the eye movement data of the participants while they were playing the virtual game. The eye tracker was an external device that was connected to a laptop through a USB interface to collect and record the participants’ eye movement data in real time. This eye tracker had a sampling frequency of 90 Hz and a gaze angle of 40 × 40 degrees. The experimental scene is shown in Figure 2.

#### 2.2.3. Procedure

The primary objective of this experiment was to explore the impact of different types of cues in virtual games on children’s JA performance. An intra-subject design was adopted in the experiment, where each group of children (ASD group and TDC group) was exposed to all three types of cues in a random order. By comparing the performance of each participant under different cues, this design effectively controls individual differences. Additionally, to address potential carryover effects, where exposure to one cue might influence the response to subsequent cues, we randomized the presentation order of the cues. This randomization was consistent among all participants to minimize systematic biases arising from the order of cue presentation and ensure equal frequencies of all cues, avoiding any single cue dominating the experiment.

The experiment was conducted in quiet classrooms at special education institutions and kindergartens. Each testing session involved two researchers, one responsible for configuring and controlling the computer equipment and the other responsible for explaining the game rules to the participants to ensure that they fully understood them. During the experiment, two independent researchers scored the participants’ key behaviors (such as responses to cues). These researchers received unified training beforehand to ensure consistency in the scoring criteria and independently recorded each participant’s behavioral performance under the different cue conditions during the experiment. Only one child participated in each experiment, and the duration of the experiment depended on the child’s speed and ability to complete the task, generally not exceeding 15 min.

The participants sat approximately 60 cm away from the computer screen. First, they calibrated the eye tracker using seven markers presented sequentially on the screen. After calibration, the participants entered the pre-test phase. During this phase, each participant underwent simulation training under three types of cues from virtual characters to familiarize themselves with the game rules and the meanings of various cues. No scores or eye movement data were recorded during this phase, with the purpose of ensuring that the participants understood the game and were prepared for the formal test.

At the beginning of the formal test, the virtual character introduced the game rules to the participants. Three LEGO bricks were randomly displayed on the computer screen, each placed in a different position. During each selection, the virtual character used a type of cue to guide the child’s gaze to the correct LEGO brick. After the introduction of the rules, the game officially began, with the virtual character immediately giving a cue while the eye tracker simultaneously started recording the participant’s eye movement data. The presentation order and frequency of each cue were randomized to ensure consistency in the experimental conditions.

The focus of this study was to evaluate participants’ responses to different types of cues and directly observe the impact of these cues on JA behavior. Therefore, we did not conduct a baseline session before the virtual character gave the cue. The participants’ gaze behavior was defined as a correct response if they maintained a gaze on the target LEGO brick for at least 2 s. If the participant correctly gazed at the target LEGO, a “√” symbol would appear above it, and the virtual character would give feedback saying, “Correct answer, you’re great!”, accompanied by a happy expression to encourage the child, and the game score would increase by 1 point. Conversely, if the participant mistakenly gazed at another LEGO, this LEGO would disappear from the screen, and the virtual character would give feedback saying, “Not this one”, accompanied by a frustrated expression, without increasing the score.

To prevent participants from feeling frustrated when they failed to make a choice, the game was designed to automatically proceed to the next selection if no correct or incorrect choice was made within 20 s. During this process, the virtual character would reuse the same cue to guide the participant to select the target, which might be in a different position from the original one, until the participant responded or the game automatically advanced to the next selection. This design ensured that the participants did not lose engagement due to inactivity for an extended period.

Throughout the entire experiment, the participants autonomously chose the correct target based on the cues given by virtual characters, while the researchers avoided providing any prompts or distractions. However, we observed that the attention of individual ASD children might be distracted, such as excessive focus on non-target objects on the screen or distractions caused by environmental factors, and these interfering behaviors might have affected the participants’ concentration and compliance. The mechanism of automatically advancing to the next round helped to mitigate these potential effects and maintained the participants’ engagement. Meanwhile, for the convenience of the subsequent data analysis, the entire experimental process was recorded using the computer’s built-in camera.

Throughout the experiment, we kept detailed records of each experimental step and reviewed and audited the process after its completion. The results showed that all experiments were conducted as planned, with no deviations or omissions identified. Through these measures, we ensured the integrity of the experimental procedures and the consistency of data collection.

#### 2.2.4. Data Analysis

Data cleaning primarily addressed the issue of missing values. The eye-tracking data were complete and had no outliers, while, for missing values in the game scores, we reviewed the videos and manually recorded the scores. We imported the data collected by the eye tracker into OGAMA 5.1 (open-source software), defined areas of interest (AOIs) based on the research questions, and analyzed eye-tracking metrics such as the fixation frequency, time spent fixating on AOIs, and fixation count. IBM SPSS 26 was then used for statistical analysis to discuss the differences between them. Additionally, to evaluate the inter-observer consistency, we calculated the level of agreement between the two independent researchers in their scoring. The Kappa coefficient (κ) was used to measure the inter-observer consistency, and the κ values were interpreted according to the following criteria: 0.61–0.80 indicates good consistency, and 0.81–1.00 indicates excellent consistency. 

The eye-tracking metrics used in the experiment included the following.

(1) Fixation Frequency. This is a common metric in measuring the degree of participants’ attention concentration on a specific area or object, and the unit is times/second. Specifically, it refers to the number of times that the participants direct their gaze towards the target area within a given time frame (per second). In this study, the fixation frequency was assessed using an eye-tracking device. This technology captures the coordinates of participants’ eye movements while they observe the game interface. By importing the recorded data into the OGAMA software, we obtained the participants’ gaze trajectories and heatmaps during gameplay, enabling us to calculate the number of fixations on specific areas, such as the correct LEGO location. After the virtual character presented Cue 3, we computed the fixation frequency by summing up the number of fixations made by participants from the cue presentation to their first gaze shift towards the correct LEGO location and dividing this sum by the total test duration (in seconds). A higher fixation frequency indicates that participants devoted more attention to these areas, potentially reflecting enhanced visual attention and information processing abilities, which are crucial aspects of JA [37]. In our game, when the virtual character gives instructions to find a specific LEGO (i.e., Cue 3), if participants can quickly and frequently shift their gaze from other areas to the correct LEGO brick, it suggests that they are actively engaged in information search and processing.

(2) Time Spent Fixating on AOIs. This refers to the duration of time that participants spend fixating within the defined AOIs, and the unit is seconds. This behavior captures the moment when the participant’s gaze falls within the designated AOI and remains stationary for more than the predefined minimum duration (e.g., 100 milliseconds) through the eye-tracking technology, representing a fixation event. Then, the total fixation duration on the AOI is calculated by accumulating the durations of all fixation events within that AOI. In our study, these AOIs correspond to specific areas on the screen, such as the face of a virtual character, a pointed LEGO, etc. For example, during a selection in the game, if the participant spends more time staring at the LEGO brick pointed to by the virtual character under the Cue 3 condition than under the Cue 1 or Cue 2 conditions, it indicates that they are highly focused on the target object under the Cue 3 condition. In the JA task, the length of time that the participants gaze at a specific target reflects their allocation and maintenance of attention to that target. Longer AOI fixation durations are generally associated with higher levels of JA [38].

(3) Fixation Count. This metric represents the total number of fixation events within a specific target area, reflecting participants’ attention distribution and switching between different regions. In this study, the operational definition of the fixation count is based on the data recorded by the Tobii 5 eye-tracking device. Specifically, a fixation is recorded when the participant’s gaze remains within the target area for more than 100 milliseconds. The total fixation count is obtained by summing all eligible fixation events throughout the test. A higher fixation count typically implies greater attention allocated to the area, underscoring its significance and importance in JA tasks [39]. In our game, when the virtual character provides cues directing participants’ attention to a specific LEGO, if the participants promptly and frequently redirect their gaze to that LEGO, it signifies successful attention guidance towards the target area. The fixation count data recorded by the Tobii 5 eye-tracker allow us to quantitatively assess participants’ attention allocation during gameplay. A significantly higher fixation count on a particular area compared to others further validates its importance in JA tasks and reflects participants’ advanced joint attention performance.

(4) Average Fixation Duration. This metric quantifies the average length of each fixation event within the target area, measured in milliseconds. In this study, the average fixation duration is calculated by dividing the total duration of all fixation events within the target area by the total number of fixations. Specifically, the Tobii 5 eye tracker records a fixation whenever a participant’s gaze remains within the target area for over 100 milliseconds. By summing the durations of all valid fixations and dividing this by the total fixation count, we obtain the average fixation duration. A longer average fixation duration suggests that participants can sustain their attention for an extended period during fixations, enabling more in-depth and detailed processing or attention to the information within the area. This is a crucial aspect in assessing the quality of JA [40]. In our game, if participants can steadily maintain their gaze on the specific target LEGO pointed out by the virtual character among multiple LEGOs for an extended period, it reflects their deep engagement and highly focused attention to that target. By recording and analyzing these fixation behaviors, we can identify which targets hold paramount importance in participants’ attention and their correlations with the directional cues provided by the virtual character.

These indicators are automatically calculated by the OGAMA software based on the collected raw eye-tracking data. Therefore, the eye-tracking results presented in this article directly reflect the output of the software analysis, without the need for further manual calculation formulas.

Given that this study addressed three research questions, we divided the data processing into three aspects. 

In the first question, we extracted the eye-tracking data of both groups under each of the three cue conditions once and imported them into the OGAMA software for processing. Since different participants required varying amounts of time to respond to a cue based on their abilities, direct comparisons of the fixation count and total fixation time between the two groups were not feasible. Instead, we compared their fixation frequencies, calculated as the ratio of the fixation count to the fixation duration.

For the second question, we investigated whether there were significant differences in the game scores (dependent variable) between children with ASD and TDC across three types of cues (independent variable). We analyzed the game scores of both ASD and TDC under each of the three cue conditions separately and determined whether there were significant differences in the game scores between the two groups.

**Figure 3:**
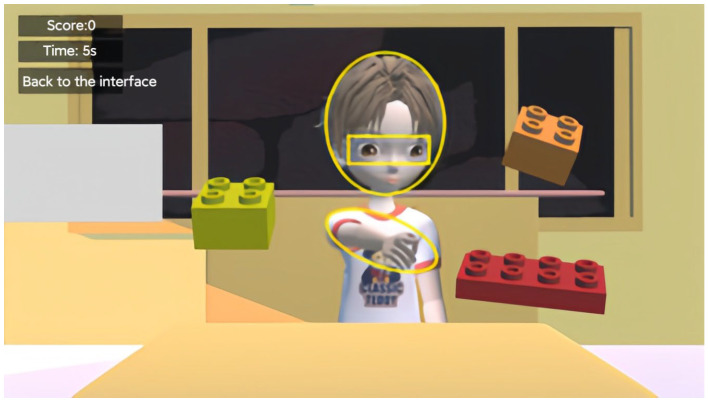
Illustration of the defined AOIs.

In the third question, we imported the eye-tracking data of ASD participants under each of the three cue conditions into the OGAMA software and defined AOIs, including the virtual character’s head, eyes, and arms (Figure 3). We analyzed the time spent fixating on these AOIs by the ASD participants across the three cue conditions and discussed the differences among them.

To analyze these three research questions, we employed a range of statistical methods, including chi-square tests, independent-samples *t*-tests, and the Kruskal–Wallis test, to examine various aspects of our data. In the following section, we will discuss the research findings related to each question separately, with a focus on the game scores and eye-tracking data. The results are presented in a structured manner to address specific aspects of the study.

## 3. Results

### 3.1. Processing of Eye-Tracking Data for ASD Children and TDC 

**Figure 4:**
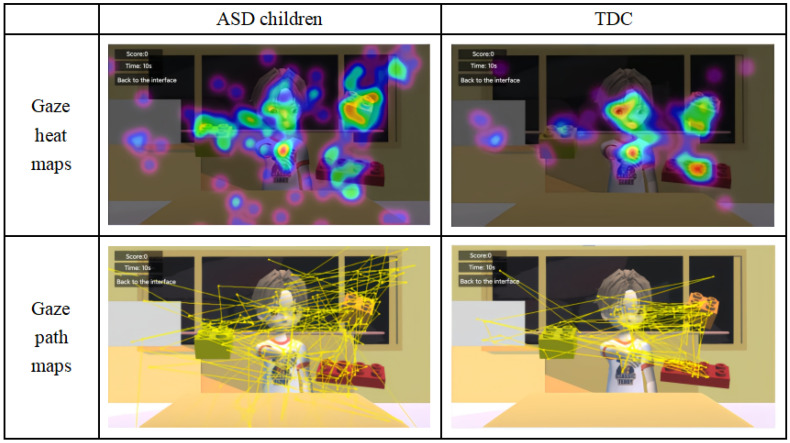
Fixation heatmaps and gaze path plots of ASD and TDC.

In the analysis of the eye-tracking data, we utilized the OGAMA software to process the fixation heatmaps and fixation path maps of both ASD and TDC (as shown in Figure 4). These visualization tools intuitively demonstrate the gaze characteristics and differences between the two groups of children under the three cue conditions. Through this comparison, we can preliminarily observe the distinct gaze patterns between the two groups.

Furthermore, we conducted a quantitative analysis of the fixation frequency for both groups, with the results presented in Table 2. The average fixation frequency for the ASD group was 0.60 (SD = 0.32), while that for the TDC group was 0.76 (SD = 0.27). To determine whether this difference was statistically significant, we performed an independent-samples *t*-test.

The results of Levene’s test for equality of variances (F = 0.74, *p* = 0.39) indicated that the variances in the fixation frequency between the two groups could be considered equal. Therefore, we adopted the *t*-test under the assumption of equal variances. The test results showed a *t*-value of 3.873 and a *p*-value less than 0.001, strongly supporting our hypothesis that there is a statistically significant difference in the average fixation frequency between TDC and ASD children, with ASD children exhibiting a significantly lower fixation frequency than TDC under the same cue condition.

### 3.2. Processing of Game Scores for ASD Children and TDC

In the virtual game related to JA, we analyzed the differences in the game scores between ASD and TDC under three different cue conditions. Table 3 shows the game scores of the two groups of children, and Figure 4 displays the accuracy rates of the two groups under each cue condition.

**Figure 5:**
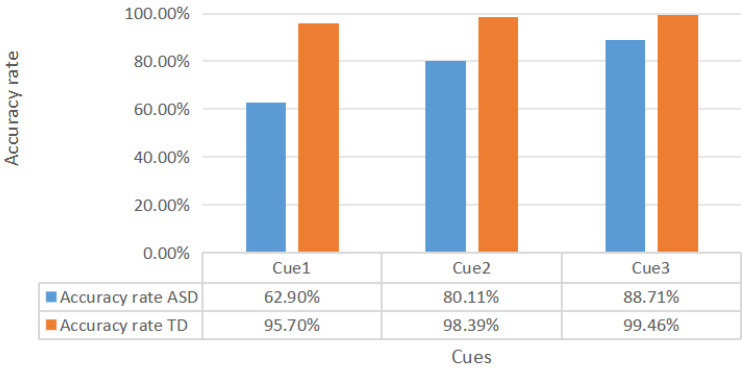
Accuracy rates of the two groups of children under different cue conditions. (accuracy rate = game score of each group under a certain cue/total score of the game under the same cue).

There were significant differences in the game scores among the ASD group children under different cue conditions. The average score under Cue 3 was the highest (4.125), while the average score under Cue 1 was the lowest (2.925). This trend is further supported by the change in the accuracy rates shown in Figure 5, where the accuracy rate of ASD children under Cue 3 is the highest.

The results of the chi-square test indicated that the type of cue had a significant impact on the game scores of the ASD group children (χ^2^ = 20.71, df = 2, *p* <0.001). Pairwise comparisons further revealed significant differences between Cue 1 and Cue 2 (χ^2^ = 4.43, df = 1, *p* <0.05), Cue 1 and Cue 3 (χ^2^ = 19.81, df = 1, *p* < 0.001), and Cue 2 and Cue 3 (χ^2^ = 5.62, df = 1, *p* < 0.05). In contrast to the ASD group, the game scores of the TDC group remained relatively stable under the different cue conditions and did not show significant differences (χ^2^ = 2.71, df = 2, *p* > 0.05). This suggests that, for TDC, different types of cues have weaker impacts on their game performance.

The results of the independent-samples *t*-test showed that, under the three cue conditions, the game scores of TDC were significantly higher than those of ASD children (M_TDC = 17.71, SD_TDC = 0.53 vs. M_ASD = 10.55, SD_ASD = 2.94; *t*(69) = 13.40, *p* < 0.001; unequal variances *t*-test: *t*(42) = 15.11, *p* < 0.001). This difference was significant in both the mean difference (7.16) and the standard error difference (0.54/0.47).

### 3.3. Processing of Eye-Tracking Data for Children with ASD under Each Cue Condition

In order to investigate the type of cue that ASD children preferred in the process of gazing at virtual games, we analyzed the number of fixations, fixation frequency, average duration of fixation, and time spent gazing at the AOIs among children in the ASD group, as shown in Table 4.

Under the three types of cues, no significant differences were observed in the number of fixations, fixation frequency, and average duration of fixation among ASD children (Kruskal–Wallis test: *p* = 0.80, *p* = 0.28, *p* = 0.10). This result indicates that ASD children did not significantly alter their basic gaze behavior patterns during the gazing process due to different cues, nor did they exhibit sensitivity or aversion to the screen.

It is worth noting that, in our experiment, the inter-observer agreement analysis showed that the Kappa coefficient between the two independent observers for all scoring items was 0.85, indicating excellent agreement. This result shows that the scoring process in the study was highly consistent.

## 4. Discussion

In this study, we employed virtual games and eye-tracking technology to investigate the effects of different cue types on JA in children with ASD and TDC. Our findings revealed significant differences in JA performance between ASD and TDC, particularly in their gaze behavior. 

Firstly, compared to TDC, children with ASD exhibit significantly reduced gaze frequencies, with more dispersed and unstable gaze patterns, indicating difficulties in processing and responding to social cues. This aligns with previous research findings that individuals with ASD have deficits in attention and social information processing [41]. This is also consistent with earlier studies that reported that children with ASD struggle with sustained attention and respond weakly to social cues such as eye contact [40,42]. In our study, TDC were more effective in following the gaze direction of the virtual characters and attending to relevant cues, displaying more stable and organized gaze behaviors. This ability to respond effectively to cues is crucial for the development of JA and broader social communication skills.

This study also found that children with ASD performed best under the integrated cue (Cue 3) that included eye contact, head turns, and pointing gestures. This preference for multi-sensory cues has also been demonstrated in other studies, which show that combining different sensory modalities can enhance attention and engagement in children with ASD [43,44]. In contrast, the play performance of TDC remained relatively stable across the different cue types, without significant differences. This disparity underscores the importance of tailoring intervention strategies to the specific needs of children with ASD and utilizing prominent multi-sensory cues to enhance their social engagement.

**Figure 6:**
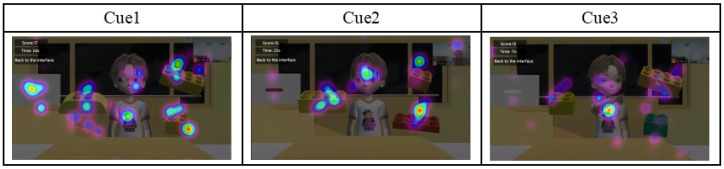
Gaze heatmaps of ASD children’s attention to three cue conditions.

Furthermore, we conducted a detailed analysis of ASD children’s preferences for specific cue types. By dividing the eyes, head, and finger pointing into AOIs, we analyzed the gaze durations for these AOIs under the three cue types and generated gaze heatmaps (Figure 6). We observed that children with ASD exhibited the longest gaze duration for the AOIs under Cue 3, exhibiting a higher visual attention bias, which supports the notion that increasing the diversity and salience of cues can improve their visual attention and engagement [45]. These findings suggest that children with ASD may benefit from intervention measures that gradually increase the complexity of social stimuli, starting from basic cues and progressively transitioning to more complex social interactions.

These findings have important implications for clinical interventions. Firstly, in educational settings, teachers can incorporate multi-sensory cues into classroom activities to better engage the attention of ASD children. For instance, during lectures, teachers can combine verbal instructions with clear pointing gestures and direct eye contact to promote attention and understanding. Studies have shown that integrating these multi-sensory cues into educational practices can significantly improve social engagement and communication behaviors in children with ASD [43,44,46,47]. Furthermore, interactive storytelling can utilize visual aids and gestures to enhance comprehension and engagement, thereby improving children’s social interaction skills.

Secondly, the atypical gaze frequency and gaze path patterns observed in children with ASD indicate the need for specialized gaze training to enhance their responsiveness to social cues. By increasing the frequency and salience of social cues and designing a phased intervention plan, we can gradually enhance their reactions to complex social cues and improve their overall JA performance. Therapists working with autistic children can design curricula that gradually increase the complexity of social cues. For example, initial sessions may focus on simple eye-tracking tasks, with the gradual addition of head movements and gestures as the child’s responsiveness improves. Early intervention programs focused on increasing the gaze duration and responsiveness to eye contact have effectively established foundational social skills in young children with ASD [48]. This phased approach allows children to develop more complex social interaction skills over time, providing them with a tailored, progressively challenging learning experience that matches their current abilities.

Moreover, utilizing eye-tracking technology to determine the AOIs and gaze preferences of children with ASD can aid in designing personalized learning and intervention content that meets their specific needs. Previous studies have shown that educational content tailored to the visual attention patterns of children with ASD can significantly improve their engagement and learning outcomes [49]. In the context of digital learning tools, developers can create programs that incorporate multi-sensory cues to capture the attention of autistic children and guide their learning process. For instance, educational games can be designed with characters using a combination of eye contact, gestures, and verbal cues to prompt children to make decisions or solve problems, simulating real-life social interactions in a controlled environment. Combining these tools with eye-tracking technology can further personalize the learning experience by adjusting the difficulty levels based on the child’s gaze patterns and attention duration, thereby providing a more adaptive and effective learning environment.

### Limitations and Future Works

Despite the valuable findings of this study, there are still some limitations. First, although we have analyzed the response patterns of children with ASD in detail in a virtual game environment, we have not tested the generalization of these results in natural environments. Therefore, it is uncertain whether these findings can be directly applied to social interactions in real life. Secondly, this study did not conduct a convergent validity test for the measurement of JA, which limits our confidence in the accuracy of the measurement method used and its consistency with other validated tools. Another limitation lies in the variability of the ASD sample, as we did not provide a detailed description of the cognitive and behavioral characteristics of the sample, which may restrict the comprehensiveness of our interpretation of the intervention effects. Finally, the design of the virtual game may not fully simulate social interaction situations in real life, which may affect the external validity of the research results and further limit their promotion in practical applications.

Future research should focus on testing the generalization of these results in natural environments to assess the performance of children with ASD in real social interaction situations. This will help to verify whether the findings obtained in the virtual environment can be translated into real-world intervention strategies. In addition, future research should include a convergent validity test for the measurement of JA to ensure the reliability and accuracy of the tools used. Moreover, we plan to use standardized assessment tools to evaluate the cognitive levels and symptom severity of ASD children. In this way, we can gain a deeper understanding of the individual differences among autistic children, as well as how these differences influence their responses to intervention measures. Further research can also improve the external validity of the experimental results by designing virtual environments that are closer to real life, so as to better understand the behavioral performance of children with ASD in diverse social situations. By pursuing these research directions, it will be possible to further optimize the intervention strategies for children with ASD and enhance the value of research in practical applications.

## 5. Conclusions

This study, by combining virtual games with eye-tracking technology, systematically explores the impact of different types of cues on JA in children with ASD and TDC for the first time. The results reveal significant differences in gaze behaviors in ASD children when facing social cues, particularly in terms of the gaze frequency and gaze path stability, consistent with previous research. These findings further validate the challenges that ASD children may face in processing complex social cues and highlight the potential benefits of integrating multiple sensory cues to enhance JA in ASD children. Unlike previous studies, this research not only analyzes children’s performance in games but also delves into their preferences for different types of cues. These findings provide a crucial theoretical basis for the further optimization of intervention strategies for ASD children. This innovative approach offers new insights for the understanding of social cognition in ASD children and lays the foundation for the development of more effective intervention strategies.

## Figures and Tables

**Table 1:** The demographics of the ASD and TDC participants.

	ASD	TDC
Number	40	31
Gender	Male: 31 (78%)	Male: 16 (52%)
	Female: 9 (22%)	Female: 15 (48%)
Age	7.05 ± 0.78	7.06 ± 0.57

**Table 2:** Descriptive statistics of fixation frequencies for both groups of children.

	ASD		TDC	
	M	SD	M	SD
Fixation Frequency	0.60	0.32	0.76	0.27

**Table 3:** Game scores of the two groups of children.

Group	Sample Size	Cue Type	Average Score	Standard Deviation	Highest Score	Lowest Score
ASD	40	Cue 1	2.925	1.421	5	0
		Cue 2	3.725	1.323	5	1
		Cue 3	4.125	1.229	6	2
TDC	31	Cue 1	5.742	0.438	6	5
		Cue 2	5.903	0.296	6	5
		Cue 3	5.968	0.167	6	5

**Table 4:** Descriptive statistics of fixation count, fixation frequency (unit: times/second), average fixation duration (unit: milliseconds), and gaze duration for AOIs (unit: seconds).

	Fixation Count		Fixation Frequency		Average Fixation Duration		Gaze Duration for AOIs	
	M	SD	M	SD	M	SD	M	SD
Cue 1	4.60	3.76	0.59	0.32	0.57	0.27	0.49	0.71
Cue 2	4.15	3.00	0.62	0.34	0.56	0.33	0.59	0.13
Cue 3	3.82	3.10	0.59	0.31	0.54	0.44	1.09	1.43

Note: All data were obtained from a sample of ASD children. For detailed statistical methods, please refer to Section 2.2.4.

## Data Availability

Due to privacy and ethical restrictions, the experimental data generated during this study are not publicly available but can be obtained from the corresponding author upon reasonable request.
